# Enrichment of a microbial community performing anaerobic oxidation of methane in a continuous high-pressure bioreactor

**DOI:** 10.1186/1471-2180-11-137

**Published:** 2011-06-16

**Authors:** Yu Zhang, Loïs Maignien, Xianxian Zhao, Fengping Wang, Nico Boon

**Affiliations:** 1 State Key Laboratory of Microbial Metabolism and School of Life Science & Biotechnology, State Key Laboratory of Ocean Engineering, Shanghai Jiao Tong University, Dongchuan Rd. 800, Shanghai, 200240, P. R. China; 2 Laboratory of Microbial Ecology and Technology (LabMET), Ghent University, Coupure Links 653, Gent, 9000, Belgium

## Abstract

**Background:**

Anaerobic oxidation of methane coupled to sulphate reduction (SR-AOM) prevents more than 90% of the oceanic methane emission to the atmosphere. In a previous study, we demonstrated that the high methane pressure (1, 4.5, and 8 MPa) stimulated *in vitro *SR-AOM activity. However, the information on the effect of high-pressure on the microbial community structure and architecture was still lacking.

**Results:**

In this study we analysed the long-term enrichment (286 days) of this microbial community, which was mediating SR-AOM in a continuous high-pressure bioreactor. 99.7% of the total biovolume represented cells in the form of small aggregates (diameter less then 15 μm). An increase of the total biovolume was observed (2.5 times). After 286 days, the ANME-2 (anaerobic methanotrophic archaea subgroup 2) and SRB (sulphate reducing bacteria) increased with a factor 12.5 and 8.4, respectively.

**Conclusion:**

This paper reports a net biomass growth of communities involved in SR-AOM, incubated at high-pressure.

## Background

Anaerobic oxidation of methane coupled to sulphate reduction (SR-AOM) is a major process determining deep-sea geochemistry and cold-seep ecosystems. First of all, it controls the atmospheric methane efflux from the ocean floor, consuming more than 90% of the methane produced in marine sediments [1]. Moreover, it fuels the deep sea ecosystem by channelling thermal generated and biogenetic methane into organic matter and carbonate. Finally, SR-AOM shapes the sea floor landscape by contributing to bicarbonate and alkalinity production, resulting in massive carbonate precipitation [2]. The overall SR-AOM reaction is:

Two groups of microorganisms are the key players in SR-AOM process: anaerobic methanotrophic archaea (ANME) with three groups (ANME-1, ANME-2 and ANME-3) and sulphate reducing bacteria (SRB) [3-6]. All ANME groups discovered so far are related clades of methanogens, while their SRB partner was always found in the same environment with or without forming spatial closely related consortia [7]. However, neither ANME nor SRB from SR-AOM active spots has been obtained in pure culture yet. The main difficulty lies on the extremely long doubling time (several months) and low growth yield (0.05 g dry weight/g carbon oxidized) of ANME and SRB from *in vitro *incubations [8-10].

To stimulate the *in vitro *SR-AOM activity and to enrich the SR-AOM community, different types of bioreactors, which can be operated at ambient/high pressure in continuous/batch mode, have been developed by different research groups [10-14]. Due to the extremely low affinity for methane (K_m _of 37 mM) and the low methane solubility at ambient pressure, high-pressure bioreactors have the advantage of permitting a higher SR-AOM activity [11,15]. Nevertheless, it is still unknown if the high-pressure bioreactor also confers advantage on biomass enrichment, and if it has an effect on selective enrichment of certain groups of ANME. Moreover, the information is lacking on the community architecture inside the high-pressure bioreactor, meaning if the microbes live as single cells or form consortia.

Through high-pressure incubation, we have obtained an enrichment originating from a Mud Volcano from the Gulf of Cadiz, performing anaerobic oxidation of methane. The SR-AOM activities at different incubation conditions have been described previously [11]. In this study, the community structure and architecture of this enrichment were investigated. The potential growth of ANME and SRB under high pressure has been evaluated.

## Results and Discussion

### Community architectural distribution

**Figure 1:**
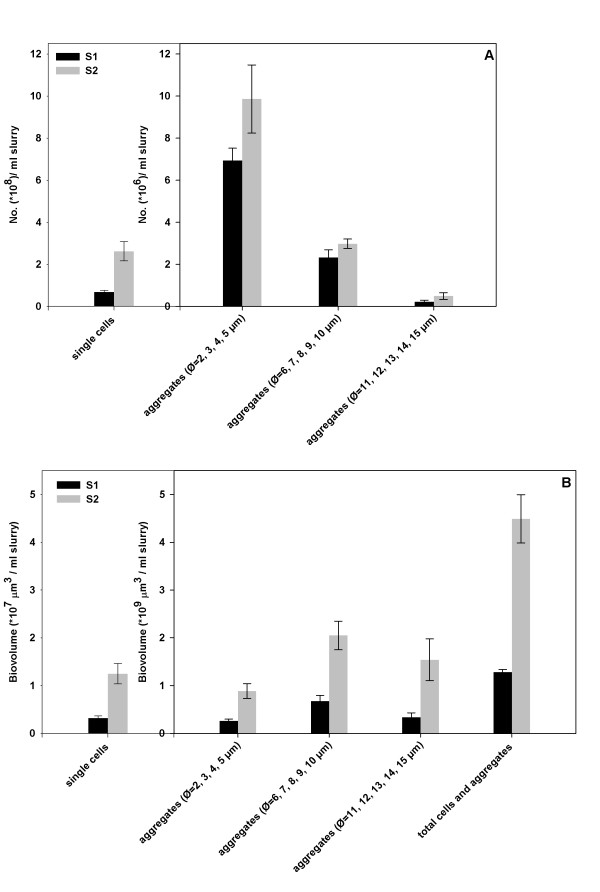
**Numbers of cells and aggregates (A) and the biovolume of cells and aggregates (B) in S1 and S2**. The average value and standard error were calculated from 4 individual staining for each sample. For each staining 50 fields of view were counted for calculation. Note that the y axe scale is different for single cells.

To access the community architectural distribution, a DAPI (4', 6-diamidino-2-phenylindole) staining was applied on the samples S1 (before high-pressure incubation) and S2 (after 286 days high-pressure incubation). Based on DAPI staining cell counts, both single cells and aggregates were commonly observed in S1 and S2. The aggregates had different sizes ranging from 2 to 15 μm in diameter (Ø). In both S1 and S2 single cells were 1-2 orders more abundant than the aggregates (Figure 1A). Among all the aggregates, the ones with diameter from 2 to 5 μm were the most abundant ones (73.35 ± 2.63% in S1 and 73.28 ± 1.75% in S2). Few spherical aggregates bigger than 15 μm were observed in S1 or S2 (less then 4 × 10^4 ^aggregates/ml slurry). For some aggregates we observed that it was dividing into two smaller spherical aggregates in both S1 and S2 (data not shown). This was also reported in another enrichment from a semi-continuous bioreactor operated under 1.4 MPa methane pressure [9]. It is an indication that these large aggregates may have reached a "critical size" during growth, which then may disintegrate into smaller aggregates for further growth.

Cell aggregates accounted for the major part of the biovolume (Figure 1B). The middle size aggregates (Ø = 6, 7, 8, 9, 10 μm) contributed for about half of the total biovolume (52.73 ± 9.04% in S1 and 47.02 ± 8.67% in S2). Although the big size aggregates (Ø = 11, 12, 13, 14, 15 μm) had very low concentrations (2.22 ± 0.74 *10^5^/ml slurry in S1 and 4.93 ± 1.56 *10^5^/ml slurry as shown in Figure 1A), they also contributed for large part of the biovolume (26.67 ± 7.83% in S1 and 33.34 ± 8.54% in S2).

### Enrichment of total biomass

The total biovolume concentration increased from (1.28 ± 0.06)*10^9 ^μm^3^/ml slurry in S1 to (4.49 ± 0.51)*10^9 ^μm^3^/ml slurry in S2 (Figure 1B). Since the reactor volume was fixed and the biomass washing out during reactor operation was negligible [11], the total biomass inside the reactor increased 2.5 times within 286 days. This reactor system was the first system that was able to accumulate total biomass while maintaining high SR-AOM activity--0.5 mmol sulfide production per day while the reactor was operated at batch mode under 8 MPa methane pressure [11]. In the systems previously reported by other authors, either only specific groups but not the total biomass was quantified [16] or there was major loss of biomass due to sampling and decay [9,10].

The biovolume data was converted into cell dry weight for a comparison with VSS (Volatile Suspended Solids) data. Taken the same assumption as described by Nauhaus et al. [9], there was about 0.2 g cell dry weight/ml biovolume in the sediment sample with SR-AOM activity. Therefore the biomass concentration in the high-pressure bioreactor increased from 0.3 (g cell dry weight/l slurry) in S1 to 0.9 (g cell dry weight/l slurry) in S2. However, this value was one order lower compared to the 8 g/l of VSS (based on weight difference between drying sample at 105°C and at 650°C) as reported by Zhang et al. [11]. One possibility is that the assumption 0.2 g cell dry weight/ml biovolume was based on analysis of two strains of small marine microorganism [9,17], which could be not representative of the cells enriched in the reactor. Another possibility would be the extracellular polymeric substances (EPS) contributed large part of VSS. For example, for granular microbial aggregates enriched in an OLAND (oxygen-limited autotrophic nitrification-denitrification) reactor, as much as 50-80% of the space occupied by bacteria was constituted of EPS [18]. For the deep-sea sediment, the presence of EPS has been reported both from *in situ *sediment and *in vitro *enrichments at different locations [9,19]. However whether the production of EPS was stimulated during high-pressure incubations and what was the mechanism behind still needs to be further investigated.

### Community structure

To identify the cells and aggregates observed under microscope, catalyzed reporter deposition fluorescence *in situ *hybridization (CARD-FISH) with probes on ANME-1, 2, 3 and SRB (Table 1) was applied on S1 and S2. Based on CARD-FISH counts, ANME-2 and SRB were the most abundant ones compared to other types of ANME, especially in the form of aggregates. Among the free-living cells, only less than 10% belonged to ANME-2 or SRB (Table 2). The number of ANME-2 aggregates accounted for 37.1 ± 6.2% of the total aggregates in S1 and 47.2 ± 8.2% in S2, while SRB accounted for 32.0 ± 6.2% of the total aggregates in S1 and 37.6 ± 5.0% in S2. However, it has to be taken into account that the CARD-FISH in this study was performed with single probe hybridization. Aggregates with ANME-2 are most probably also containing SRB as well, because they tend to live closely and form consortia [7,9]. No ANME-1 was detected in S1 and S2. About 2% of ANME-3 was detected in the aggregates (Table 2).

**Table 1:** Primers and probes used in this study.

Name (labelling)	Sequence (5' to 3')	Positions	Specificity	References
PCR primers				
Arch-21f	TTC CGG TTG ATC CYG CCG GA	21-40	Archaea	[28]
Arch-958r	YCC GGC GTT GAM TCC AAT T	958-976	Archaea	[28]
27f	AGA GTT TGA TCC TGG CTC AG	27-46	Eubacteria	[29]
1492r	GGT TAC CTT GTT ACG ACT T	1492-1510	Eubacteria	[30]

CARD-FISH probes				
ANME1-350	AGT TTT CGC GCC TGA TGC	350-367	ANME-1 archaea	[4]
EelMS932	AGC TCC ACC CGT TGT AGT	932-949	ANME-2 archaea	[4]
ANME3-1249	TCG GAG TAG GGA CCC ATT	1250-1267	ANME-3 archaea	[31]
ANME3-1249H3	GTC CCA ATC ATT GTA GCC GGC	1229-1249	Helper probe for ANME3-1249	[32]
ANME3-1249H5	TTA TGA GAT TAC CAT CTC CTT	1268-1288	Helper probe for ANME3-1249	[32]
DSS658	TCC ACT TCC CTC TCC CAT	658-685	*Desulfosarcina *spp., *Desulfofaba *spp., *Desulfococcus *spp., *Desulfofrigus *spp.	[33]

**Table 2:** Community composition based on CARD-FISH analysis

Samples	% of cell count ^1^	% of aggregate count ^1^	% of biovolume^1^
S1			
ANME-1	Below detection limit^2^	Below detection limit^2^	Below detection limit^2^
ANME-2	8.2 ± 3.0	37.1 ± 6.2	13.4 ± 4.2
ANME-3	0.1 ± 0.1	2.1 ± 1.4	1.5 ± 1.5
SRB	2.9 ± 1.5	32.0 ± 6.2	22.7 ± 5.3

S2			
ANME-1	Below detection limit^3^	Below detection limit^3^	Below detection limit^3^
ANME-2	2.5 ± 2.0	47.2 ± 8.2	50.4 ± 15.9
ANME-3	0.1 ± 0.1	0.8 ± 0.7	2.4 ± 1.8
SRB	0.8 ± 0.4	37.6 ± 5.0	60.6 ± 5.5

^1 ^The average value and standard error were calculated based on 50 fields of view on each hybridization. No ANME-1 cell or aggregate was observed based on our method. ^2 ^Detection limit of 4 × 10^4 ^cells/ml slurry. ^3 ^Detection limit of 9 × 10^4 ^cells/ml slurry

**Figure 2:**
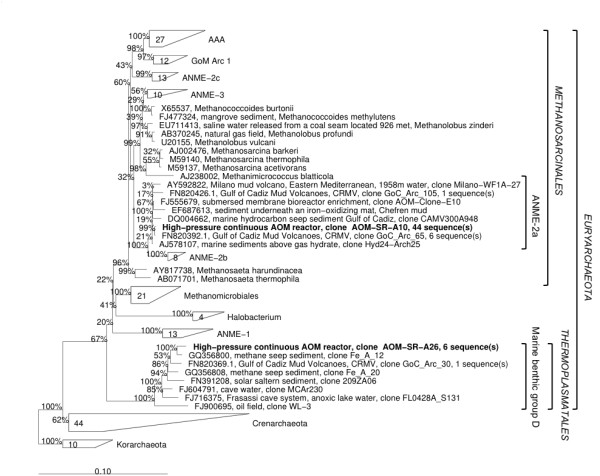
**Phylogenetic tree showing the affiliations of archaeal 16S rRNA gene sequences detected from S2 to selected reference sequences**.

**Figure 3:**
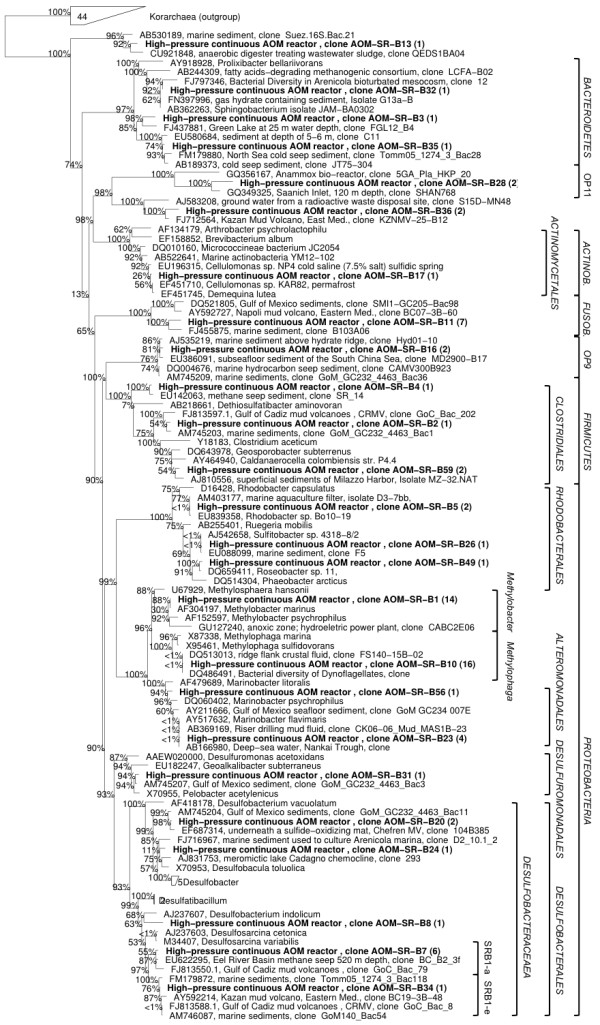
**Phylogenetic tree showing the affiliations of bacterial 16S rRNA gene sequences detected from S2 to selected reference sequences**.

The CARD-FISH result showed that a large part of biomass in S1 and S2, especially single cells, did not belong to ANME or SRB. There was growth of other unknown microbes within a mixed community of ANME/SRB. Therefore a clone library analysis was performed on S2 to approach to the complete archaeal and bacterial communities. Archaeal community had extremely low diversity, where ANME-2a and MBG-D (marine benthic group D) were the only two groups of archaea detected. ANME-2a was the dominant, which accounted for 88% of the archaeal community (Figure 2). No 16S rRNA gene from ANME-3 was detected. The absence of ANME-3 in the archaeal clone library was contradictory to CARD-FISH result. The size of the clone library was not large enough to detect the rare ANME-3 or the hybridization experiment may have led to mis-hybridization, thus giving false positive signal. Dissimilar from archaeal community, the bacterial community was highly diverse (Figure 3). Gammaproteobacteria (43%) were the most dominant followed by the Deltaproteobacteria (17%), which includes the SRB. Among total bacteria population in S2, 8% was belonging to SEEP-SRB1a subgroup of *Deltaproteobacteria*, which were found to be specifically associated with ANME-2a in other enrichments mediating SR-AOM process [20]. Most of the Gammaproteobacteria found in the community were closely related to *Methylophaga *sp. and *Methylobacter *sp., which are known to use reduced one-carbon compounds, such as methane, methanol or dimethylsulphide [21]. The presence of such bacteria in our anaerobic reactor is intriguing since methane and sulphate were the only electron donor and acceptor supplied. The presence and even production of sulphide (sulphide concentration increased up to 0.5 mM everyday in the reactor) was an indication of anaerobic condition inside the reactor. However we cannot exclude the possibility of a limited amount of dissolved oxygen in the reactor influent, which could explain the presence of aerobic. Further tests need to show if these Gammaproteobacteria are playing an important active role in the reactor.

### Enrichment of ANME-2 and SRB

CARD-FISH results showed that percentages of ANME-2 and SRB biovolume increased from 13.4 ± 4.2% and 22.7 ± 5.3% in S1 to 50.4 ± 15.9% and 60.6 ± 5.5% in S2 (Table 2). By combining with the total biovolume data from DAPI staining (Figure 1B), the biovolume of ANME-2 in S1 was:

(1.28*10^9 ^μm^3^/ml slurry) * 13.4% = 1.7*10^8 ^μm^3^/ml slurry

The biovolume of ANME-2 in S2 was:

(4.49*10^9 ^μm^3^/ml slurry) * 50.4% = 2.3*10^9 ^μm^3^/ml slurry

Therefore after 286 days incubation, the ANME-2 population increased for 12.5 times. Following the same method of calculation, the SRB population increased for 8.4 times after 286 days incubation in this high-pressure bioreactor. The populations of ANME-2 and SRB both increased faster than the total biomass, which indicated that ANME-2 and SRB were selectively enriched in the system. This selective enrichment of ANME-2 and SRB was another proof that the incubation condition inside this high-pressure bioreactor was favourable for SR-AOM community.

To our knowledge, this is the first report on the enrichment of SR-AOM community under high methane pressure, although potential growth of ANME-1, ANME-2 and SRB has been reported in other engineered systems at ambient or low methane pressures (Table 3). The different inocula showed different doubling times. When ANME-1 and ANME-2c were incubated in continuous flow bioreactors under ambient methane partial pressure, ANME-1 had doubling time of 1.1 months while ANME-2c had doubling time of 1.4 months [16]. High methane partial pressure appeared to have advantage on stimulating the growth of ANME. In the experiment of Krüger *et al*. [22], the methane-dependent uptake of ^15^N-NH_4 _by AOM community dominated by ANME-1 was higher at 1.5 MPa methane pressure than at ambient methane pressure. If we assume the ANME-2a cells in our system were following a logarithmic growth curve, a doubling time of 2.5 months can be estimated based on ANME-2 biovolume in S1 and S2, which is shorter than the result (3.8 months of doubling time of ANME-2a from an ambient pressure bioreactor) obtained by Meulepas *et al*. [10]. The increase of energy gained from SR-AOM process by increasing methane pressure may favour the biomass growth [8,22]. Continuous flow also stimulated growth: ANME-2a/2c had longer doubling time in a fed-batch bioreactor (7.5 months) than in continuous flow bioreactors (1.4-3.8 months) (Table 3).

**Table 3:** Comparison of doubling times of ANME in different enrichment systems

Sediment origin	ANME group	Methane pressure	Operational mode	Doubling time (months)	Reference
Monterey Bay	ANME-1	Ambient	Continuous flow	1.1	[16]
Gulf of Mexico	ANME-1	1.5 MPa	Batch	2-3.4	[22]
Eckernforde Bay	ANME-2a	Ambient	Continuous flow	3.8	[10]
Monterey Bay	ANME-2c	Ambient	Continuous flow	1.4	[16]
Hydrate Ridge	ANME-2a/2c and SRB consortia	1.4 MPa	Fed-batch	7.5	[9]

## Conclusions

After 286 days incubation in a simulated cold seep environment under high methane pressure, ANME-2 and SRB in the sediment from Captain Arutyunov Mud Volcano were enriched. Based on biovolume calculation, the populations of ANME-2 and SRB increased for 12.5 times and 8.4 times. Within total biomass volume, 99.7% was accounted from aggregates. Therefore the incubation condition apparently favoured the cells to form aggregates, especially in small size (2<Ø≤5 μm), rather than to live as single cells. No aggregate bigger than 15 μm in diameter was observed; they apparently divided after reaching a critical size. Based on the 16S rRNA gene clone library, the archaeal diversity was low, and contained only ANME-2 (88%) and MBG-D (12%). In contrast, the bacterial community was highly diverse.

## Methods

### Incubation condition

In a previous study, the sediment sample originally from Captain Arutyunov Mud Volcano (Gulf of Cadiz, North East Atlantic) was diluted 12 times with artificial sea water medium and incubated in a continuous high-pressure bioreactor at 15°C [11]. This bioreactor system was a simulator for cold seep ecosystems, where sulphate and high-pressure methane were supplied. Because the high apparent affinity for methane (37 mM) in SR-AOM reaction and low dissolubility of methane in seawater (1.3 mM at 15°C at ambient pressure), it is necessary to supply high pressure methane to obtain high concentration of dissolved methane which can be directly used by microorganisms for high *in vitro *SR-AOM activity [11]. During this research, the reactor was operated in a fed-batch mode or a continuous mode. When it was in fed-batch mode, the methane pressures were switched between 1, 4.5 and 8 MPa. When it was in continuous mode, the methane pressure was either 1 or 8 MPa and the flow rate was 0.1 ml/min (HRT 100 hours). The SR-AOM activities under different operational conditions have been described previously [11]. To take a slurry sample, the incubation vessel was open under a nitrogen atmosphere and manually stirred to make the slurry sample homogeneous. The slurry samples before (S1) and after (S2) 286 days incubation were fixed in 4% formaldehyde and stored at 4°C for cell staining. Additional slurry from S2 was stored at -20°C for DNA extraction and clone library analysis.

### Cell and aggregates quantification

To assess the number and the size of cells and aggregates, DAPI (4', 6-diamidino-2-phenylindole) staining was performed on S1 (after 2000 times dilution) and S2 (after 5600 times dilution). Subsequently, the samples were filtrated onto a circular GTTP polycarbonate filter (0.2 μm, Millipore, Germany) with a diameter of 2.5 cm. The number of cells (or aggregates) was quantified under a microscope (Zeiss, Carl Zeiss Microimaging GmbH, Germany) at 1,000 times magnification. The diameter of a single cell was assumed as 0.45 μm, which is the average size of ANME and SRB cells [3,9]. The diameters of the aggregates were measured according to a reference scale bar built in the eyepiece of the microscope. The biovolume was calculated assuming that both cells and aggregates have spherical shapes. For each sample, 4 individual staining were applied. For each staining 50 fields of view were counted for calculation.

### Cell and aggregates identification

In order to evaluate which type of ANME and SRB were present and enriched in the reactor, catalyzed reporter deposition fluorescence *in situ *hybridization (CARD-FISH) was applied on S1 and S2. The slurry samples were embedded onto GTTP filters. The filters were incubated in methanol with 0.15% H_2_O_2 _for 30 min at room temperature before washed with water and ethanol and dried. For each sample, 2 filters were prepared. One was incubated in lysozyme solution (10 mg/ml in 0.05 M EDTA, pH 8.0; 0.1 M Tris-HCl, pH 8.0) for 15 min at 37°C to achieve permeablilization of bacterial cells, and another one was incubated in Proteinase K solution (15 μg/ml in MilliQ water) for 3 min at room temperature to achieve permeabilization of achaeal cells. Afterwards the filters were cut into 4 pieces. Each piece was for hybridization with one probe (Table 1). The hybridization was performed according to the protocol previously described [23]. After hybridization, the filter was stained with DAPI to target all cells present on the filter.

During CARD-FISH, a few steps of washing the filter may cause the loss of cells and aggregates. It was assumed that all types of cells or aggregates were washed out in the same ratio. Therefore the percentage of ANME or SRB among the total cells did not change after washing. For each hybridization, cells and aggregates in 50 fields of view were analyzed under microscope. For each field, both probe staining and DAPI staining were counted to quantify the concentration of ANME-1 (or ANME-2, ANME-3 and SRB) among total biomass.

For a more detailed investigation on the microbial community, the archaeal and bacterial 16S rRNA gene clone libraries were performed on S2 according to protocol previously described [24,25] with the primers listed in Table 1. For archaeal library, 56 clones were obtained while 50 clones were randomly picked for sequencing. For bacterial library, 110 clones were obtained while 100 clones were picked for sequencing. The sequences were compared with their best match in NCBI to classify their phylogenetic group (Additional file 1, Table S1). To calculate the percentage of each phylogenetic group into total archaeal/bacterial community, the number of clones within one phylogenetic group was divided by the number of sequenced clones within archaeal/bacterial library. All the sequences described in the paper have been deposited in the databases of GenBank, under accession numbers HQ405602 to HQ405741. The archaeal hpylogenetic tree has been constructed by Maximum Likelihood based on 212 sequences using the online RaxML tool at http://phylobench.vital-it.ch/raxml-bb/index.php[26]. The bacterial phylogenetic tree has been constructed using the online maximum likelihood tool at http://www.atgc-montpellier.fr/phyml/[27].

## Authors' contributions

YZ carried out the incubation and DAPI staining, participated in CARD-FISH and drafted the manuscript. LM carried out the CARD-FISH and participated on the sequence analysis. XZ and FW carried the clone libraries and sequence analysis. NB conceived of the study, and participated in its design and coordination and helped to draft the manuscript. All authors read and approved the final manuscript.

## Supplementary Material

Additional file 1 **Table S1**. Clones obtained from archaeal and bacterial 16S rRNA libraries. Indicating the clones name, best match, similarity and the groups they belong to. Click here for file
