# Supplementary material for: Enrichment of a microbial community performing anaerobic oxidation of methane in a continuous high-pressure bioreactor
Source: BMC Microbiol. 2011 Jun 16;11:137. doi: 10.1186/1471-2180-11-137 (PMC3142483; doi:10.1186/1471-2180-11-137)
Supplement: Additional file 1 — Table S1. Clones obtained from archaeal and bacterial 16S rRNA libraries. Indicating the clones name, best match, similarity and the groups they belong to. [file 1471-2180-11-137-S1.DOC]

Additional file 1

Table S1. Clones obtained from archaeal and bacterial 16S rRNA libraries.

| Clones | Best match | Similarity | Groups belonging to |
| --- | --- | --- | --- |
| Archaea | | | |
| AOM-SR-A4(16)^*^ | clone BS-K-H6 | 99% | ANME2a |
| AOM-SR-A8 | clone BS-K-H6 | 99% | ANME2a |
| AOM-SR-A13(3) | clone AOM-Clone-C9 | 99% | ANME2a |
| AOM-SR-A49 | clone BS-K-H6 | 98% | ANME2a |
| AOM-SR-A6(13) | clone BR34ARC_C05 | 99% | ANME2a |
| AOM-SR-A29 | clone BS-K-H6 | 99% | ANME2a |
| AOM-SR-A16(2) | clone BS-K-H6 | 99% | ANME2a |
| AOM-SR-A14(7) | clone AOM-Clone-C9 | 98% | ANME2a |
| AOM-SR-A2(2) | clone KZNMV-25-A2 | 99% | MBGD |
| AOM-SR-A24 | clone KZNMV-25-A2 | 99% | MBGD |
| AOM-SR-A12 | clone KZNMV-25-A2 | 99% | MBGD |
| AOM-SR-A40(2) | clone KZNMV-25-A2 | 99% | MBGD |
| Bacteria | | | |
| AOM-SR-B95 | Uncultured bacterium clone KZNMV-5-B68 | 99% | Actinobacteria |
| AOM-SR-B17 | Uncultured bacterium clone LC33 | 97% | Actinobacteria |
| AOM-SR-B5 | Rhodobacter sp. Bo10-19 | 98% | Alphaproteobacteria Rhodobacter |
| AOM-SR-B54 | Rhodobacter sp. Bo10-19 | 98% | Alphaproteobacteria Rhodobacter |
| AOM-SR-B49 | Rhodobacteraceae bacterium JAM-AL0110 | 99% | Alphaproteobacteria Rhodobacteraceae |
| AOM-SR-B26 | Sulfitobacter dubius strain KMM 3554 | 99% | Alphaproteobacteria Sulfitobacter |
| AOM-SR-B35 | clone: JT75-304 | 98% | Bacteroidetes Cytophaga |
| AOM-SR-B32 | Sphingobacteria bacterium JAM-BA0302 | 98% | Bacteroidetes Sphingobacteria |
| AOM-SR-B3 | Uncultured Cytophagales clone Hyd89-72 | 93% | Bacteroidetes Sphingobacteriales |
| AOM-SR-B19 | Subsaxibacter sp. ZS4-19 | 95% | Bacteroidetes Subsaxibacter |
| AOM-SR-B29 | Subsaxibacter sp. ZS4-19 | 96% | Bacteroidetes Subsaxibacter |
| AOM-SR-B74 | Uncultured Chlorobi bacterium clone MVP-4 | 95% | Chlorobi |
| AOM-SR-B68 | Uncultured bacterium PENDANT-36 | 95% | Chloroflexi |
| AOM-SR-B6 | Uncultured clone Eel-36e1H1 | 98% | Deltaproteobacteria |
| AOM-SR-B7 | Uncultured clone Eel-36e1H1 | 98% | Deltaproteobacteria |
| AOM-SR-B18 | Uncultured clone Eel-36e1H1 | 98% | Deltaproteobacteria |
| AOM-SR-B44 | Uncultured clone Eel-36e1H1 | 98% | Deltaproteobacteria |
| AOM-SR-B45 | Uncultured clone Eel-36e1H1 | 98% | Deltaproteobacteria |
| AOM-SR-B58 | Uncultured clone Eel-36e1H1 | 98% | Deltaproteobacteria |
| AOM-SR-B100 | Uncultured bacterium clone LARIS_37-01E05 | 99% | Deltaproteobacteria |
| AOM-SR-B99 | Uncultured bacterium clone: ODP1230B18.24 | 97% | Deltaproteobacteria |
| AOM-SR-B34 | Uncultured bacterium clone KZNMV-5-B1 | 99% | Deltaproteobacteria |
| AOM-SR-B20 | Uncultured Desulfobacteraceae bacterium | 95% | Deltaproteobacteria Desulfobacteraceae |
| AOM-SR-B24 | Delta proteobacterium JS_SRB50Hy | 96% | Deltaproteobacteria Desulfobacteraceae |
| AOM-SR-B83 | Uncultured Desulfosarcina sp. clone SB4_53 | 99% | Deltaproteobacteria Desulfobacteraceae |
| AOM-SR-B89 | Uncultured Desulfobacteraceae bacterium | 96% | Deltaproteobacteria Desulfobacteraceae |
| AOM-SR-B8 | Uncultured clone HMMVBeg-12 | 95% | Deltaproteobacteria Desulfobacteraceae |
| AOM-SR-B31 | Pelobacter acetylenicus strain WoAcy1 | 96% | Deltaproteobacteria Pelobacter |
| AOM-SR-B78 | Uncultured bacterium clone 107B250 | 98% | Epsilonproteobacteria |
| AOM-SR-B2 | Uncultured bacterium clone KZNMV-30-B76 | 93% | Firmicutes |
| AOM-SR-B4 | Uncultured bacterium clone GoM GC234 616E | 96% | Firmicutes |
| AOM-SR-B61 | Uncultured bacterium clone SC172 | 99% | Firmicutes |
| AOM-SR-B87 | Desulfosporosinus auripigmenti strain OREX-4 | 97% | Firmicutes Clostridia |
| AOM-SR-B88 | Alcanivorax venustensis isolate MARC4P | 99% | Gammaproteobacteria Alcanivorax |
| AOM-SR-B96 | Legionella beliardensis strain Montbeliard A1 | 89% | Gammaproteobacteria Legionella |
| AOM-SR-B23 | Marine bacterium HP15 | 98% | Gammaproteobacteria Marinobacter |
| AOM-SR-B37 | strain LMG 24048 | 99% | Gammaproteobacteria Marinobacter |
| AOM-SR-B50 | Marine bacterium HP15 | 98% | Gammaproteobacteria Marinobacter |
| AOM-SR-B56 | Marinobacter sp. MARC4V | 97% | Gammaproteobacteria Marinobacter |
| AOM-SR-B65 | Marinobacter sp. Ws22 | 99% | Gammaproteobacteria Marinobacter |
| AOM-SR-B86 | Marine bacterium HP15 | 99% | Gammaproteobacteria Marinobacter |
| AOM-SR-B85 | Marinobacter isolate MARC4F | 99% | Gammaproteobacteria Marinobacter |
| AOM-SR-B1 | Methylobacter sp. BB5.1 | 99% | Gammaproteobacteria Methylobacter |
| AOM-SR-B12 | Methylobacter sp. BB5.1 | 99% | Gammaproteobacteria Methylobacter |
| AOM-SR-B14 | Methylobacter sp. BB5.1 | 99% | Gammaproteobacteria Methylobacter |
| AOM-SR-B15 | Methylobacter sp. BB5.1 | 99% | Gammaproteobacteria Methylobacter |
| AOM-SR-B27 | Methylobacter sp. BB5.1 | 99% | Gammaproteobacteria Methylobacter |
| AOM-SR-B33 | Methylobacter sp. BB5.1 | 99% | Gammaproteobacteria Methylobacter |
| AOM-SR-B38 | Methylobacter sp. BB5.1 | 99% | Gammaproteobacteria Methylobacter |
| AOM-SR-B41 | Methylobacter sp. BB5.1 | 99% | Gammaproteobacteria Methylobacter |
| AOM-SR-B53 | Methylobacter sp. BB5.1 | 99% | Gammaproteobacteria Methylobacter |
| AOM-SR-B71 | Methylobacter sp. BB5.1 | 99% | Gammaproteobacteria Methylobacter |
| AOM-SR-B72 | Methylobacter sp. BB5.1 | 99% | Gammaproteobacteria Methylobacter |
| AOM-SR-B73 | Methylobacter sp. BB5.1 | 99% | Gammaproteobacteria Methylobacter |
| AOM-SR-B81 | Methylobacter sp. BB5.1 | 99% | Gammaproteobacteria Methylobacter |
| AOM-SR-B90 | Methylobacter sp. BB5.1 | 99% | Gammaproteobacteria Methylobacter |
| AOM-SR-B9 | Methylophaga sp. V4.MO.19 | 99% | Gammaproteobacteria Methylophaga |
| AOM-SR-B10 | Methylophaga sp. V4.MO.19 | 99% | Gammaproteobacteria Methylophaga |
| AOM-SR-B21 | Methylophaga sp. V4.MO.19 | 99% | Gammaproteobacteria Methylophaga |
| AOM-SR-B30 | Methylophaga sp. V4.MS.17 | 99% | Gammaproteobacteria Methylophaga |
| AOM-SR-B39 | Methylophaga sp. V4.MO.19 | 99% | Gammaproteobacteria Methylophaga |
| AOM-SR-B40 | Methylophaga sp. V4.MO.19 | 98% | Gammaproteobacteria Methylophaga |
| AOM-SR-B43 | Methylophaga sp. V4.MO.19 | 99% | Gammaproteobacteria Methylophaga |
| AOM-SR-B46 | Methylophaga sp. V4.MO.19 | 99% | Gammaproteobacteria Methylophaga |
| AOM-SR-B47 | Methylophaga sp. V4.MO.19 | 99% | Gammaproteobacteria Methylophaga |
| AOM-SR-B48 | Methylophaga sp. V4.MO.19 | 99% | Gammaproteobacteria Methylophaga |
| AOM-SR-B57 | Methylophaga sp. V4.MO.19 | 99% | Gammaproteobacteria Methylophaga |
| AOM-SR-B101 | Uncultured Methylophaga sp. clone IAFJAsip1 | 99% | Gammaproteobacteria Methylophaga |
| AOM-SR-B75 | Methylophaga sp. V4.MO.19 | 99% | Gammaproteobacteria Methylophaga |
| AOM-SR-B84 | Uncultured Methylophaga sp. clone IAFJAsip1 | 98% | Gammaproteobacteria Methylophaga |
| AOM-SR-B93 | Uncultured Methylophaga sp. clone IAFJAsip1 | 99% | Gammaproteobacteria Methylophaga |
| AOM-SR-B91 | Uncultured Methylophaga sp. clone IAFJAsip1 | 99% | Gammaproteobacteria Methylophaga |
| AOM-SR-B69 | isolate MZ-32.NAT | 96% | Gram Positive Low G+C |
| AOM-SR-B59 | isolate MZ-32.NAT | 96% | Gram-positive low G+C |
| AOM-SR-B16 | Uncultured bacterium clone KZNMV-30-B24 | 98% | JS1 |
| AOM-SR-B67 | Uncultured bacterium clone KZNMV-30-B24 | 99% | JS1 |
| AOM-SR-B11 | Uncultured bacterium clone B050C03 | 96% | OP1 |
| AOM-SR-B22 | Uncultured bacterium clone B050C03 | 96% | OP1 |
| AOM-SR-B42 | Uncultured bacterium clone B050C03 | 96% | OP1 |
| AOM-SR-B52 | Uncultured bacterium clone B050C03 | 96% | OP1 |
| AOM-SR-B55 | Uncultured bacterium clone B050C03 | 95% | OP1 |
| AOM-SR-B80 | Uncultured bacterium clone B050C03 | 96% | OP1 |
| AOM-SR-B82 | Uncultured bacterium clone B050C03 | 96% | OP1 |
| AOM-SR-B98 | clone: ODP1251B13.14 | 98% | OP8 |
| AOM-SR-B13 | clone QEDN3BB02 | 94% | Planctomycetes |
| AOM-SR-B92 | Uncultured bacterium clone SK27B-16 | 90% | Planctomycetes |
| AOM-SR-B36 | Uncultured bacterium clone KZNMV-25-B12 | 99% | TG1 |
| AOM-SR-B79 | Uncultured bacterium clone KZNMV-25-B12 | 99% | TG1 |
| AOM-SR-B28 | Uncultured bacterium clone SHAN768 | 98% | unknown |
| AOM-SR-B76 | Uncultured bacterium clone SHAN768 | 98% | unknown |

^*^ the numbers in bracks indicate the number of sequences with 100% similarities.
